# Complete Genome Sequence of Human Norovirus GII.Pe-GII.4 Sydney from the United States

**DOI:** 10.1128/genomeA.00159-17

**Published:** 2017-04-13

**Authors:** Zhihui Yang, Jan Vinjé, Michael Kulka

**Affiliations:** a Division of Molecular Biology, Office of Applied Research and Safety Assessment, Center for Food Safety and Applied Nutrition, U.S. Food and Drug Administration, Laurel, Maryland, USA; b Division of Viral Diseases, Centers for Disease Control and Prevention, Atlanta, Georgia, USA

## Abstract

We report here the first near-complete genome sequence (7,551 nucleotides) of a human norovirus GII.Pe-GII.4 Sydney variant, detected in a stool sample from an outbreak on a cruise ship in 2013.

## GENOME ANNOUNCEMENT

Human noroviruses have been identified as the primary cause of acute viral gastroenteritis worldwide (1). Among the more than 30 genotypes that cause disease in humans, GII.4 viruses have been reported to cause the majority of norovirus infections worldwide (2, 3). New GII.4 variants have emerged every 2 to 3 years, displacing previous dominant strains (4). GII.Pe.-GII.4 Sydney variant emerged in late 2012 (5) and accounted for 53% of the outbreaks in the United States from September to December 2012 (6). In 2015, a new GII.4 Sydney variant (GII.P16-GII.4 Sydney [KX907727]) emerged, which in the 2016–2017 season is replacing GII.Pe-GII.4 Sydney as the predominant outbreak strain (http://www.cdc.gov/norovirus/reporting/calicinet/data.html). However, there is no complete genome sequence of GII.Pe-GII.4 Sydney from the United States available in GenBank. We report here the first near-complete genome sequence of a human norovirus GII.Pe-GII.4 Sydney, detected in a stool sample associated with an outbreak on a cruise ship.

Viral RNA was extracted from the supernatant of a 10% (wt/vol) norovirus-positive stool sample in phosphate-buffered saline using a QIAamp Viral RNA mini kit (Qiagen), followed by library construction using a TruSeq mRNA library prep kit (Illumina) and sequencing on the MiSeq platform (Illumina). Data were analyzed using CLC Genomics Workbench (CLC bio). One contig covering the norovirus genome sequence was assembled from 5,165,549 reads containing an average coverage of 66,490×.

The near-complete (lack of 5′ untranslated region [UTR] of 5 nucleotides [nt]) genome sequence was 7,551 nt in length and contained (i) three open reading frames [ORFs]: ORF1, ORF2, and ORF3 of 5,094, 1,623, and 807 nt in length, respectively; and (ii) a 3′ UTR of 48 nt in length. The sequence could be typed as GII.Pe-GII.4 Sydney by using the Norovirus Genotyping tool (7). A BLAST search confirmed that this sequence shares 99% (7,448/7,540) nucleotide similarity with the prototype GII.4 Sydney 2012 strain (GenBank accession no. JX459908.1).

Adding an additional near-complete genome sequence of a GII.Pe-GII.4 Sydney variant provides an additional reference sequence for phylogenetic and evolutionary studies aimed at better understanding the mutation frequency of GII.4 variant strains.

### Accession number(s).

The genome sequence of the GII.Pe-GII.4 Sydney variant/USA has been deposited in GenBank under the accession number KY486271.
